# Association of glucocorticoid receptor gene polymorphism and occupational stress with hypertension in desert petroleum workers in Xinjiang, China

**DOI:** 10.1186/s12881-018-0688-4

**Published:** 2018-12-13

**Authors:** Ning Tao, Hua Ge, Wenfeng Wu, Hengqing An, Jiwen Liu, Xinjuan Xu

**Affiliations:** 1 Department of Public Health, Xinjiang Medical University, Urumqi, 830011 China; 2 Clinical post-doctoral mobile stations, Xinjiang Medical University, Urumqi, 830011 China; 3 The First Affiliated Hospital, Xinjiang Medical University, Urumqi, 830011 China; 4 Public Health and Preventive medicine post-doctoral mobile station, Xinjiang Medical University, Urumqi, 830011 China

**Keywords:** Desertpetroleum workers - occupational stress, Hypertension - GR gene polymorphism

## Abstract

**Background:**

The aim of this study was to investigate the occupational stress and hypertension in desert petroleum workers in Xinjiang, and to analyze the association of occupational stress and glucocorticoid receptor (GR) gene polymorphism with the presence of hypertension.

**Methods:**

Using cluster sampling, 1280 desert petroleum workers of 3 petroleum fields in Xinjiang Karamay were randomly selected as the target group for this study. According to the inclusion criteria, a total of 1080 workers were included as the baseline for this study. We followed these workers for 2 years to investigate their occupational stress and hypertension. The polymorphism of GR gene was detected by polymerase chain reaction-restriction fragment length polymorphism. We applied appropriate statistical methods to analyze the association of occupational stress and glucocorticoid receptor (GR) gene polymorphism with the presence of hypertension.

**Results:**

After 2 years of follow-up, there were 995 desert petroleum workers in the queue. The study showed that the incidence of hypertension in desert petroleum workers were 19.4%. Compared with the baseline data, the level of occupational stress increased, and with the increase of occupational stress, the incidence of hypertension was gradually increasing. A positive relationship was observed in the GR BCL1 gene polymorphisms and hypertension. Relative to the CC genotype, carries of the GG genotype had a significantly higher risk of hypertension (OR = 2.830). With the combination of genotype CG and GG, carries of CG and GG increased the risk of hypertension (adjusted OR = 2.238, 95%CI:1.104–4.940). There was no significant association between GR G678S gene polymorphisms and hypertension.

**Conclusion:**

GR gene polymorphism and occupational stress of desert petroleum workers were important risk factors for hypertension.

## Background

With the rapid development of the global economy and the pace of life gradually accelerated, the social competition intensified, making the professional population to bear enormous social psychological stress. To adapt to the development of modern society, people’s lifestyles and work patterns have changed, occupational stress is particularly prominent, and become one of the most important public health problems in the world [1]. Occupational stress is widespread, especially in highly stressful professions. For example, medical workers, police officers, teachers, railway workers, drivers, oil workers, et al. [2–7]. A study suggested that moderate occupational stress was essential and beneficial to health, it could arouse the enthusiasm of workers, inspire potential, improve work efficiency [8]. However, excessive occupational stress adversely affected the physiology, psychology and behavior of the laborers, which might cause various physical diseases, anxiety and occupational injuries [9].

Hypertension is a most common chronic disease that threatens human health caused by the interaction of environmental, social and genetic factors, and is the most important risk factor for cardiovascular and cerebrovascular disease [10]. Risk factors for the occurrence of hypertension are numerous, mainly high-salt diet, obesity, smoking, drinking, genetic, etc. [11]. With the transformation of medical model, the researchers gradually realized that a person’s health level, not only with a variety of natural factors, but also affected by the social psychological factors [12]. An animal experiment showed that stress could raise blood pressure in rats, increase their heart rate, limit their activity and show depression [13]. A cross-sectional survey of 1035 managers demonstrated that high pay-low return work patterns would increase the occupational stress of workers and significantly Related to hypertension [14]. Studies have shown that chronic stress can cause blood pressure fluctuations and as a potential risk factor for hypertension [15]. Researchers conducted a five-year follow-up survey of 1394 white-collar workers, and the results revealed that men who had been exposed to high-intensity jobs for a long time were more likely to suffer from high blood pressure and that women had long-term work under high pay-low return conditions would lead to increased systolic blood pressure [16]. While dealing with the sudden occupational injuries caused by occupational stress, desert petroleum workers should also pay attention to adverse social psychological pressure on the physical and mental health.

Occupational stress of physiological reactions related to the body’s various systems and organs, mainly neuroendocrine system changes [17]. when the body reacts to the stressor, the hypothalamic-pituitary-adrenal (HPA) cortical systemis activated to release a large number of adrenal cortical hormones, causing increased activity of adrenal cortical cells, resulting in more glucocorticoids(GCs). Glucocorticoids(GCs) are the key hormones in the body to adapt to stress, and play a biological role through binding glucocorticoid receptor (GR) [18]. Studies have shown that variation in GR at rs41423247 and rs258747, stressful life events, social support, and the number oftraumatic events were each separately associated with the risk for Post-traumatic stress disorder (PTSD) [19]. A study has shown that glucocorticoids(GCs) canregulate blood pressure by regulating vascular reactivity [20, 21]. An animal experiment has found that low dose of GR agonist can increase the blood pressure of double transgenic mice [22]. The epidemiological survey indicated that GR gene polymorphism could cause blood pressure fluctuation, blood glucose and lipid metabolism abnormalities, and related to cardiovascular disease [23]. Another study among European-Americans showed that GR gene variantis closely related to the blood pressure [24]. Lin S et al. reported that the interaction between CG/GG genotype and occupational stress in GR BCL1 locus can increase the risk of primary hypertension in railway workers [5].

Many of production oil fields in Karamay are in the remote and desolate gobi desert region, with harsh natural conditions, dry weather, summer heat and cold winter. Desert petroleum workers not only affected by extreme weather, besides, they have heavy tasks, long working hours and irregular shift work. Studies have shown that unsuitable working conditions can damage the physical and mental health of the occupational population [25]. Furthermore, studies indicated that occupational stress is related to hypertension [26, 27]. At present, most of the studies on petroleum workers are cross-sectional surveys, with fewer cohort studies. Therefore, this study carried out a 2-year follow-up study of desert petroleum workers to investigate their occupational stress and hypertension, and to analyze the association of occupational stress and glucocorticoid receptor (GR) gene polymorphism with the presence of hypertension. It is important to remission the occupational stress and control the morbidity of hypertension of desert petroleum workers. It is of great significance to promote the health of desert petroleum workers and improve their professional quality of life.

## Methods

### Study population

The implementation of the study has been through the Xinjiang Medical University Ethics Committee (XJMU # 2012005). This study began in July 2014 and ended in July 2016, with a prospective follow-up of 2 years. 1280 workers of 3 petroleum administrations in Karamay city of Xinjiang were randomly selected as a target group for the study. The sampling method is as follows: There are 5 factories in the 3 petroleum administrations, each with 4 teams and a total of 20 teams. These 20 teams are numbered, and randomly selected 10 teams according to random random number table method. A total of 1280 desert petroleum workers from 10 teams were used as research objects. In accordance with the inclusion criteria, a total of 1080 desert petroleum workersentered the baseline queue.All subjects were informed of consent. Inclusion criteria: (1) Employees aged from 18 to 60 years old who; (2) Worked on more than one year; (3) In the past week, no taking tricyclic antidepressant, dexamethasone, estrogens and glucocorticoids drugs; (4) No organic disease, mental illness, hypertension, hereditary hypertension and other genetic history; (5) Those who are willing to cooperate with the collection of blood samples, after the investigators inform the purpose and significance of the study.

### Research contents and steps

During the follow-up period, 1080 desert petroleum workers conducted a unified medical examination every year at the Central Hospital of Karamay City. At the same time, the investigators instructed them to complete the “Occupational Health Status Survey for Petroleum Workers”, which was exactly the same as the baseline survey. Occupational stress as an exposure factor, investigating the incidence of hypertension from the beginning of the study to the end of follow-up. The “Occupational Health Status Survey for Petroleum Workers” mainly includes general demographic data,Occupational Stress Inventory and blood pressure measurements. General demographic characteristics covers the sex, age, ethnicity, educational level, job tenure, job title, shift situation, smoking history and history of drinking. Blood collection was performed by the medical workers of the center hospital of Karamay, after the blood collection was completed, the plasma and serum were separated and stored in a refrigerator at − 20 °C. At the end of follow-up, the blood samples of hypertensive patients were screened and the polymorphism of GR gene was detected by polymerase chain reaction-restriction fragment length polymorphism. Finally, according to the results to analyze the association of occupational stress and glucocorticoid receptor (GR) gene polymorphism with the presence of hypertension.

### Occupational stress

We used the Occupational Stress Inventory (revised)(OSI-R) to assess the occupational stress of the desert petroleum workers [28]. The scale is composed of three subscales: Occupational Roles Questionnaire (ORQ), Personal Strain Questionnaire (PSQ), and Personal Resources Questionnaire (PRQ) respectively. The OPQ is used to test the occupational stress factors of the subjects. The subscale included six subkeys: role overload, role insufficiency, role ambiguity, role boundary, responsibility and working condition, each subkey contained 10 topics, a total of 60 topics. The PSQ was used to assess workers’ occupational stress levels. The subscale contained four subkeys: vocational strain, psychological strain, interpersonal strain, and physical strain, each subkey contained 10 topics, a total of 40 topics. The PRQ was used to evaluate the ability of workers to cope with occupational stress. The subscale contains four subkeys: relaxation, self-care, social support and rational coping, each subkey contained 10 topics, a total of 40 topics. The options for each topic in this scale are: “1” = no, “2” = less, “3” = sometimes, “4” = more often, “5” = often, the workers selected “no” for 1 point, selected “less” for 2 points, selected “sometimes” for 3 points, selected “more often” for 4 points, selected “often” for 5 points. The higher OPQ score, indicating more occupational stress factors. The higher PSQ score, the more severe the degree of occupational stress. The higher PRQ score, the stronger ability to cope with occupational stress [29]. The Occupational stress groups were divided into three levels based on PSQ. According to the general population of China, original scores for each dimension of PSQ were linearly converted to standard T scores (mean = 50, standard deviation = 10) [30]. T scores ≥70 for the high occupational stress group, T scores are between 60 and 69 for the moderate occupational stress group, and T scores ≤59 for the low occupational stress group [30].In our study, the content validity was deemed acceptable, as the Cronbach’s alpha was 0.862 and the split-half reliability was 0.751. Therefore, the questionnaire can be used to assess the level of occupational stress of desert petroleum workers.

### Blood pressure measurement

When workers unified medical examination, the medical workers of the center hospital measured blood pressure for them. Participants were asked to sit for at least 10 min. The right upper arm blood pressure were measured twice by Omron calibrated electronic sphygmomanometer for each subject, and the average of the two measurements was the final result. According to the Guidelines for Prevention and Treatment of Hypertension in China (2010), the systolic blood pressure ≥ 140 mmHg and the diastolic blood pressure ≥ 90 mmHg diagnosed as hypertension [31].

### DNA sampling and genotyping

Venous blood samples were obtained as part of the health examination and were anonymized. Samples were collected in EDTA-containing tubes from each participant following a 12 h fast. The samples were placed in the centrifuge for 3 min, at 800 rpm to separate serum and plasma, and stored at − 20 °C. The polymorphism of the GR gene was detected by polymerase chain reaction-restriction fragment length polymorphism.

### GR BCL1 and GR G678S genotype analysis

Polymerase chain reaction-restriction fragment length polymorphism (PCR-RFLP) method was used to identify polymorphisms in both locus (GRBCL1 and GR G678S). The upstream primer sequence for the GR BCL1 is 5’-AGAGCCCTATTCTTCAAACTG-3′ and the downstream primer sequence is 5’-GAGAAATTCACCCCTACCAAC-3′, with a length of 418 bp.The PCR amplification system was determined and PCR amplification was carried out according to the amplification conditions of the GR BCL1. The PCR conditions were predenatured at 94 °C for 5 min, then denatured at 94 °C, annealed at 57 °C and extended at 72 °C, each step lasted 35 s, after 32 cycles,with a final extension for 10 min at 72 °C, and finally stored at 4 °C. The genotypes of GR BCL1 sites are CC genotype (fragment length is 418 bp), CG genotype (fragment length is 418 + 267 + 151 bp) and GG genotype (fragment length is 267 + 151 bp). The alleles are C and G. The upstream primer sequence for the GR G678S is 5’-CACAGTGAGACCCTATCTATC-3and the downstream primer sequence is 5’-AAACATACTTTGTCCCAGAG-3′, with a length of 303 bp. The PCR conditions were predenatured at 94 °C for 5 min, then denatured at 94 °C, annealed at 50 °C and extended at 72 °C, each step lasted 45 s, after 32 cycles,with a final extension for 5 min at 72 °C, and finally stored at 4 °C. The genotypes of GR G678S sites are CC genotype (fragment length is 303 bp), CT genotype (fragment length is 303 + 204 + 99 bp) and TT genotype (fragment length is 204 + 99 bp). The alleles are C and T. Following enzymatic digestion, PCR products were resolved on 2.5% agarose gel electrophoresis and visualized by ethidium bromide staining.

### Statistical analysis

The data were input using EpiData version 3.1 database (The EpiData Association, Odense, Denmark), and the results were processed by SPSS20.0 statistical software (SPSS Inc., Chicago, IL, USA).The concentration trend and the discrete trend of the normal distribution continuous variables are described by mean and standard deviation. In this study, the occupational stress scores in baseline queue and the follow-up queue were normal and the variance were equal. Therefore, t-test was used to analyze the changes in scores of occupational stress in baseline queue and the follow-up queue. Categorical variables were expressed by percentage. The chi-square test was used to analyze the occurrence of hypertension of the different demographic characteristics among desert petroleum workers, explore the relationship between occupational stress levels and BCL1 and G678S of GR gene. Multivariate logistic regression analysis was used to explain the effects of occupational stress factors and BCL1 genotype on hypertension. In the Multivariate Logistic regression analysis, the entry probability is 0.05 and the exclusion probability is 0.1. In this study, all analyzes were bilateral test, the test level was α = 0.05. There was a statistically significant difference when *P* < 0.05.

## Results

### Demographic characteristics of population

A total of 1080 workers were included in the cohort, 85 workers were censored when the follow-up ended, and 995 petroleum workers were followed up. The participants comprised 545 men and 450 women, of whom, 143 were ≤ 30 years of age and 852 were > 30 years of age, 798 were of Han nationality and 197 were of ethnic minorities, 297 had a length of service ≤15 years and 698 had a length of service > 15 years, 381 had fixed day shifts and 614 had variable shifts, 359 had junior occupation titles, 201 had secondary occupation titles, and 435 had senior occupation titles, 406 had a college education and 589 had an undergraduate or greater education, 321 were smokers, and 440 were drinkers. The cohort retention rate was 92.13%.

### Comparison of baseline and follow - up queuing occupational stress scores

At the end of the follow-up, the scores for occupational stress were higher than the baseline scores. We found significant differences in role overload, role insufficiency, role ambiguity, role boundaries, working conditions, vocational strain, interpersonal strain, relaxation, self-care, social support, and rational coping (*P* < 0.05). There was no significant difference between the sense of responsibility, psychological strain, and physical strain (*P* > 0.05; Table 1).

**Table 1:** Comparison of baseline and follow-up queuing occupational stress scores

Items	Baseline	Follow-up	*t*	*P* value
Role overload	25.92 ± 6.98	27.12 ± 6.98	4.664	< 0.001
Role insufficiency	28.98 ± 7.76	30.31 ± 7.64	4.648	< 0.001
Role ambiguity	30.96 ± 8.06	31.67 ± 6.99	2.645	< 0.001
Role boundary	26.59 ± 6.64	26.83 ± 6.46	0.955	< 0.001
Responsibility	24.75 ± 6.71	24.76 ± 7.22	0.051	0.960
Working condition	29.60 ± 7.89	31.84 ± 7.89	6.334	< 0.001
Vocational strain	23.90 ± 6.35	25.10 ± 6.25	4.927	< 0.001
Psychological strain	25.75 ± 6.96	25.91 ± 6.63	0.620	0.535
Interpersonal strain	25.93 ± 6.15	26.79 ± 5.78	3.750	< 0.001
physical strain	24.96 ± 6.37	25.16 ± 6.33	0.835	0.404
Relaxation	26.48 ± 7.02	28.17 ± 6.87	6.457	< 0.001
Self-care	27.77 ± 26.90	28.99 ± 6.74	4.894	< 0.001
Social support	30.42 ± 8.59	32.14 ± 8.14	8.634	< 0.001
Rational coping	28.38 ± 7.75	29.71 ± 7.12	4.781	< 0.001

### Follow-up population hypertension incidence

At the end of follow-up, we confirmed the presence of hypertension in 193 workers. The incidence rate was 19.4%, with 19.63% in male and 19.11% in female workers. The systolic blood pressure level was (147.30 ± 10.567)mmHg, and the diastolic blood pressure level was (74.28 ± 13.077)mmHg in hypertensive patients. The systolic blood pressure and diastolic blood pressure levels in non-hypertensive patients were (114.56 ± 10.703)mmHg and (66.76 ± 12.692)mmHg, respectively. The incidence of hypertension was significantly different between the smoking group and the non-smoking group, and between the alcohol consumption group and the no-alcohol consumption group (*P* < 0.05). There was no significant difference in sex, age, ethnicity, job tenure, shift situation, title, and educational levels (*P* > 0.05; Table 2).

**Table 2:** Hypertension incidence according to subject characteristics

Characteristics	*N*	Hypertension(n)	Incidence rate (%)	*χ* ^*2*^	*P* value
Sex					
Male	545	107	19.63	0.179	0.672
Female	450	86	19.11		
Age(years)					
≤40	243	34	13.99	6.008	0.014
> 40	752	159	21.14		
Ethnic					
Han ethnic	798	153	19.17	0.000	0.999
Ethnic minority	197	40	20.30		
Job tenure(years)					
≤15	297	56	18.89	0.291	0.589
> 15	698	137	19.63		
Shift situation					
Day shift	381	73	19.16	0.360	0.548
Shift work	614	120	19.54		
Title					
Primary	359	67	18.66	5.561	0.062
Secondary	201	39	19.40		
Senior	435	87	20.00		
Education level					
College and below	406	79	19.46	0.033	0.855
Undergraduate and above	598	114	19.35		
Smoking					
Yes	321	71	22.12	6.678	0.009^*^
No	674	122	18.10		
Drinking					
Yes	440	97	22.05	8.945	0.003^*^
No	555	96	17.30		

^*^p < 0.05

### Association between occupational stress and hypertension

At the end of follow-up, the numbers of hypertensive patients in the low, moderate, and high occupational stress groups were 34persons, 72persons and 87persons, and the cumulative incidence of hypertension was 17.6%, 37.3%, and 45.1% respectively. There were statistically significant differences in the incidence of hypertension between different occupational stress groups (*P* < 0.05; Table 3).

**Table 3:** Association between occupational stress level and hypertension

Job stress level	Hypertensive		Non-hypertensive	
	*N*	%	*n*	%
Low job stress	34	17.6	121	15.1
Medium job stress	72	37.3	438	54.6
High job stress	87	45.1	243	30.3
*χ*^*2*^ = 11.293 *P* = 0.004				

### Association between job strain and hypertension

In order to analyze the influence factors of hypertension in desert petroleum workers, hypertension as the dependent variable, statistically significant variables were used as independent variables in single factor analysis, using stepwise regression to analyze.

Logistic regression analyses assessed the relationship between job strain and hypertension (Table 4). At the end of follow-up, the risk of hypertension in the group with high role insufficiency was 2.16 timehigher (95% CI, 1.63–2.97) than that in the low group (low at cohort initiation and high at the end of follow-up). The risks of hypertension in the group with high vocational strain was 1.62 times higher (95% CI, 1.22–2.02) than that in the low group (low at cohort initiation and high at end of follow-up). The risk of hypertension in the group with high physical strainwas 2.32 times higher (95% CI, 1.78–2.989) than that in the low group (low at cohort initiation and high at the end of follow-up). High rational coping was a protective factor against job strain (RR = 0.51, 95% CI: 0.32–0.82).

**Table 4:** Association between occupational stress and hypertension

Occupational stress	*β*	SE	*Wald*	*P*	*RR*(95%*CI*)
role insufficiency	0.337	0.120	7.833	0.005^*^	2.158(1.634-2.967)
vocational strain	0.408	0.126	10.201	0.001^*^	1.623(1.217-2.024)
physical strain	0.821	0.123	40.575	< 0.001^*^	2.318(1.781-2.987)
rational coping	−0.464	0.122	15.463	< 0.001^*^	0.512(0.319-0.824)

*RR*, unadjusted relative risk; *CI*, confidence interval

^*^p < 0.05

### Hardy-Weinberg genetic equilibrium test

For the experiment, 179 hypertensive patients were randomly selected as the case group, and 179 non-hypertensive patients were used as the control group according to the 1:1 matching principle. In the case group, the systolic blood pressure was (141.49 ± 14.333) mmHg, and the diastolic blood pressure was (74.72 ± 12.052) mmHg. In the control group, the systolic blood pressure was (117.52 ± 10.029) mmHg and the diastolic blood pressure was (68.70 ± 11.739) mmHg. There was no significant difference in the distribution of actual value and expected value between the three genotypes of GR BCL1 locus and the two genotypes of GR G678S in the case group and the control group (*P >* 0.05), indicating that their distributions of actual value and expected value are consistent with Hardy-Weingerg’s law of genetic equilibrium (Table 5).

**Table 5:** GR Hardy-Weinberg genetic equilibrium test

Genetic locus	Genotype	Case group		*χ* ^*2*^	*P*	Control group		*χ* ^*2*^	*P*
		actual value	expected value			actual value	expected value		
GR BCL1	CC	10	19.12	5.163	0.076	25	31.01	1.727	0.422
	CG	97	78.76			99	86.99		
	GG	72	81.12			55	61.01		
GR G678S	CC	160	160.53	0.030	0.862	157	157.83	0.026	0.871
	CT	19	17.97			22	20.51		

### GR gene polymorphisms are related to hypertension (Table 6)

For GR BCL1, χ^2^ analysis revealed differences for genotype and allele distribution between the case group and the control group (*P* < 0.05). For GR G678S, there were no differences between the case group and the control group (*P* > 0.05). It is suggested that GR BCL1 is related to the susceptibility of hypertension, and GR G678S is not related to the susceptibility to hypertension, so GR G678S will not be used for subsequent analysis.

**Table 6:** Association between GR gene polymorphisms and hypertension

Genetic locus	Genotype	Case group(%)	Control group(%)	*χ* ^*2*^	*P* value
GR BCL1	CC	10 (5.6)	25 (14.0)	8.725	0.013^*^
	CG	97(54.2)	99(55.3)		
	GG	72 (40.2)	55 (30.7)		
	C	117 (32.7)	149 (41.6)	6.125	0.013^*^
	G	241 (67.3)	209 (58.4)		
GR G678S	CC	160 (89.4)	157 (87.7)	0.248	0.619
	CT	19 (10.6)	22 (12.3)		
	C	339 (94.7)	336 (93.9)	0.233	0.629
	T	19 (5.3)	22 (6.1)		

^*^p < 0.05

### Association between GR BCL1 and hypertension (Table 7)

Logistic regression analysis was used to assess the effect of GR BCL1 genotypes polymorphisms on hypertension, expressed as odds ratio (OR) and 95% confidence interval. A significantly positive relationship GR BCL1 gene polymorphisms and hypertension was observed. Relative to the CC genotype, carries of the CG and GG genotypes had a significantly higher risk of hypertension (OR = 2.204, 2.975 respectively). After all potential risk factors were adjusted, there was no statistically significant difference between the CG genotype and hypertension. With the combination of genotype CG and GG, carries of CG and GG increased the risk of hypertension (adjusted OR = 2.238, 95%CI:1.104–4.940).

**Table 7:** Association between GR BCL1 and hypertension

Genetic locus	Genotype	*OR(95%CI)*	*OR* ^*^ *(95%CI)*
GR BCL1	CC	1	1
	CG	2.204(1.028~ 4.725)^*^	1.970(0.876~ 4.431)
	GG	2.975(1.349~ 6.564)^*^	2.830(1.206~ 6.640)^*^
	CG + GG	2.479(1.180~ 5.207) ^*^	2.238(1.014~ 4.940)^*^

*OR*, odds ratio; *CI*, confidence interval

OR*, adjusted odds ratios (*OR*) for body mass index, smoking, passive smoking, alcohol consumption, and occupational stress level

^*^p < 0.05

### Analysis of GR BCL1 variant

**Fig. 1:**
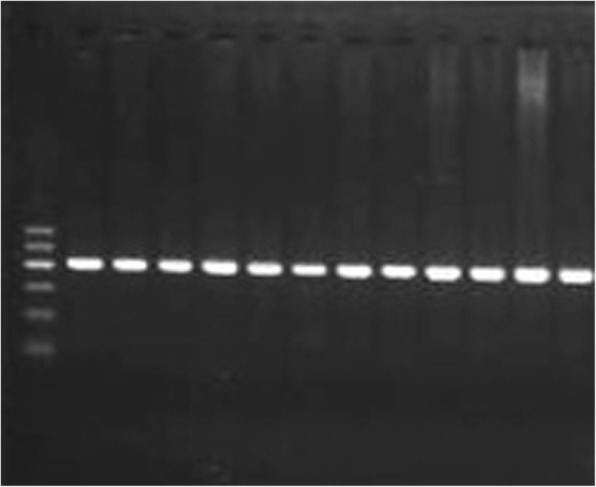
GR BCL1. PCR products showing 418 bp amplicon (Lanes 1–12). Lane M: 100 bp ladder

**Fig. 2:**
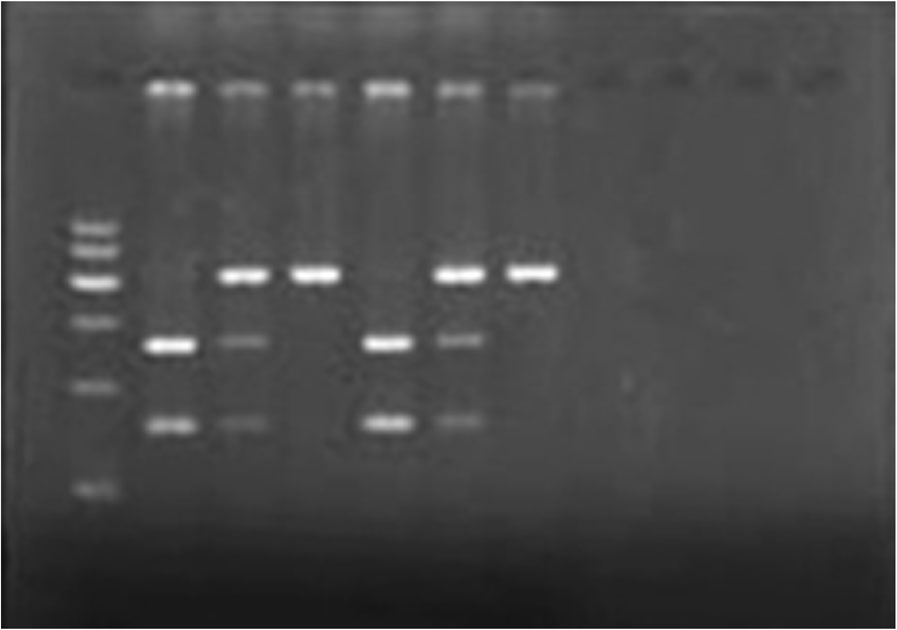
GR BCL1. Lanes 1, 4: GG genotype; Lanes 2, 5: CG genotype; Lanes 3, 6: CC genotype; Lane M: 100 bp ladder

GR genotyping (BCL1) was performed by PCR followed by RFLP. The presence of product was verified on a 2% agarose gel stained with ethidium bromide, a band of 418 bp was observed as shown in Fig. 1. The PCR product was digested by Bcl I restriction enzyme and visualized by 2.5% agarose gel. A band of 418 bp was observed for the CC genotype, two bands of 267, 151 bp was observed for the GG genotype while three bands of 418, 267, 151 bp were observed for the heterozygous genotype CG as shown in Fig. 2.

### Analysis of GR G678S variant

**Fig. 3:**
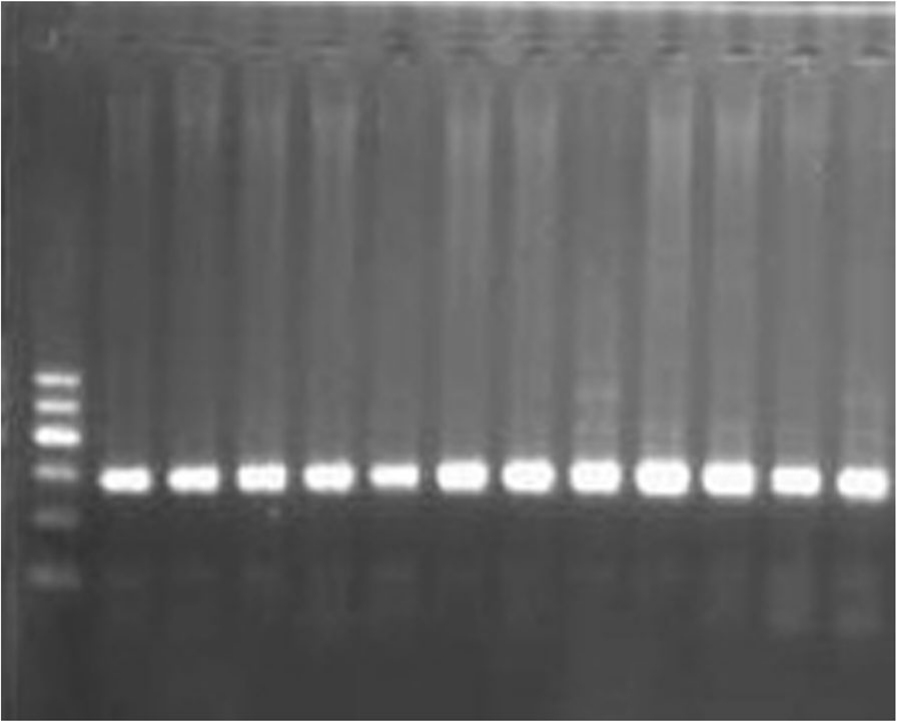
GR G678S. PCR products showing 303 bp amplicon (Lanes 1–12). Lane M: 100 bp ladder

**Fig. 4:**
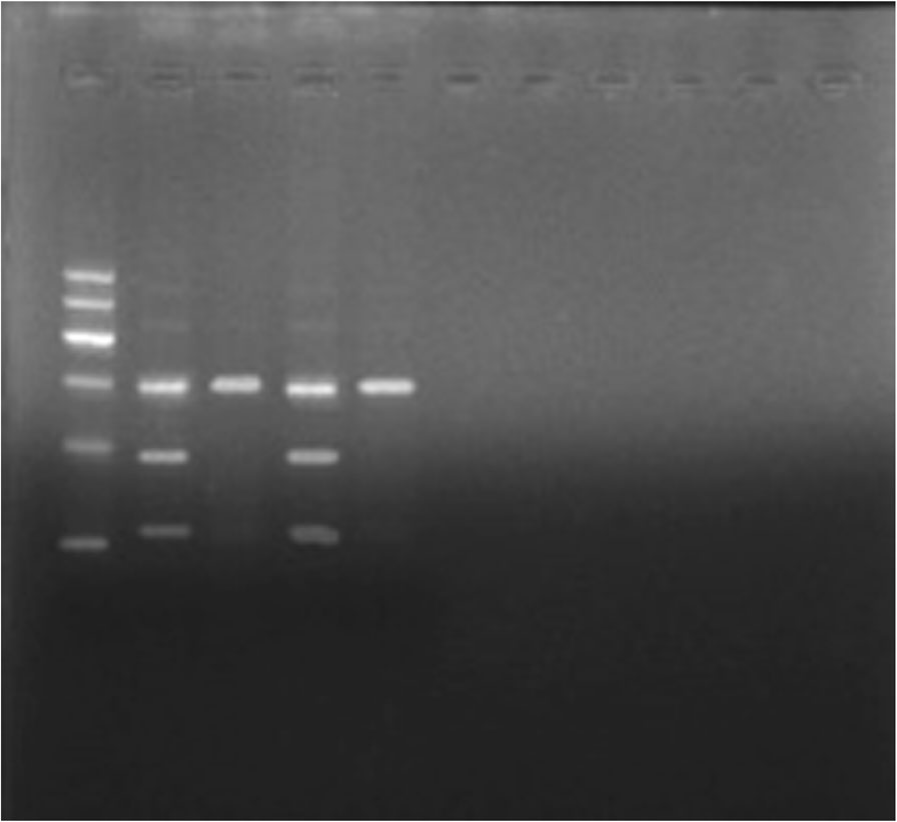
GR G678S. Lanes 2, 4: CC genotype; Lanes 1, 3: CT genotype; Lane M: 100 bp ladder

GR genotyping (G678S) was performed by PCR followed by RFLP. The presence of product was verified on a 2% agarose gel stained with ethidium bromide, a band of 303 bp was observed as shown in Fig. 3. The PCR product was digested by Fork I restriction enzyme and visualized by 2.5% agarose gel. A band of 303 bp was observed for the CC genotype, two bands of 204, 99 bp was observed for the TT genotype while three bands of 303, 204, 99 bp were observed for the heterozygous genotype CT as shown in Fig. 4.

## Discussion

Due to the special nature of the desert petroleum workers, they are often exposed to a variety of occupational hazards, these harmful factors through the various organs into the body, so that the function of a variety of organs are seriously damaged [32]. A study in Finland found that unsuitable working conditions damage the physical and mental health of employees [33].Desert petroleum workers have arduous tasks, long working hours, mostly shift work, and insufficient sleep. Khajehnasiri [34] showed that shift workers had a high level of stress and depressive symptoms. An unreasonable task allocation scheme can affect the judgment of workers, prolong working time, reduce work efficiency [35]. Harmonious interpersonal relationships can promote mental health [36]. However, Interpersonal strain make employees lack of work enthusiasm, job satisfaction decline, resulting in lower productivity levels [37]. Tensions can be alleviated through a variety of ways. Studies have suggested that social support played an important role in coping with occupational stress [38]. Yu J [39] indicated that people with higher social support have rich resources and are more capable of responding to stress. At the same time, to maintain a positive and optimistic attitude can make the body relax and reduce the incidence of disease [40]. Exercise is an effective means of reducing stress [41], studies declared that regular exercise not only reduced the incidence of cardiovascular disease, but also relieved mental stress [42].

Hypertension is the most common cardiovascular disease, with high morbidity, high disability and high mortality [43]. Several studies founded that smoking and drinking increase the incidence of hypertension, consistent with this study [44]. In recent years, more researchers have found that social psychological factors play a key role in the pathogenesis of hypertension [45], occupational stress has become one of the culprits of hypertension [46]. A meta-analysis of the incidence of hypertension in professional population, occupational stress is an important risk factor for hypertension [47]. The more severe the occupational stress, the higher the incidence of hypertension, consistent with the results of Lin-bo Fan [48]. Based on the above findings, workers should pay more attention to their physical condition, stop smoking and drinking, finally reduce the incidence of hypertension from the source. Meanwhile, the employer should lay emphasis on the psychological changes of workers, ease the pressure in time, and create an optimistic and positive working atmosphere.

To further analyze the relationship between occupational stress and hypertension, the results pointed out that the role insufficiency, vocational strain and physical strain may increase the incidence of hypertension, rational coping may reduce the incidence of hypertension. With the rapid development of oil extraction technology, mechanical automation equipment is gradually replacing the manual operation, the work pattern from manual labor gradually transformed into a combination of manual labor and mental work. In order to adapt to this change, workers need to improve oil extraction technology, learn new technologies and knowledge, participate in various training and assessment. These requirements increase the workload of workers, cause lead to role insufficiency and vocational strain, and increase the risk of Cardiovascular [49]. Desert petroleum workers are labor intensive, lacking physical exercise, with an irregular diet and sleeping, frequent exposure to various occupational hazards, resulting in varying degrees of physical strain, and high blood pressure [50]. Rational coping is a protective factor in reducing hypertension, consistent with the results of Lian [51]. The above findings provide clues to the influencing factors of hypertension and provide strategies for the prevention and treatment of hypertension.

In the correlation analysis of hypertension and the GR gene, GR BCL1 was associated with a susceptibility to hypertension, and a C → G mutation of the GR BCL1 allele increased the risk of hypertension. GCs are an important class of steroid hormones in the body, affecting the lipid metabolism, glucose metabolism, bone metabolism, and brain function, and have the physiological function of inhibiting immune response and inflammation [52, 53]. Van Moorsel [54] showed that GR BCL1 gene polymorphism will increase the risk of hypertension and cardiovascular disease, and confirmed that the GG genotype at BCL1 is closely related to hypertension. A C → G mutation on GR gene exon 2 results in the production of serine instead of aspartic acid. The phosphorylation of the serine residue can increase the transcriptional activation of the GR reaction gene, and cause the sensitivity of the peripheral tissue to GCs to increase the inhibition of endothelial cells, to produce prostaglandins, nitric oxide and other vasodilatory factors. Along with the increase in vasoconstriction, the action of smooth muscle on vasoconstriction also increased, thus inducing hypertension [55]. Kaur et al. [56] have suggested that mutations in the GR BCL1 allele C → G are associated with a blood pressure increase, consistent with the results of this study.

The scale system used in this study is a dependable, scientifically validated tool with good reliability. There are some limitations to this study. As this was a prospective cohort study, there was an attrition bias during follow-up. Measurement of blood pressure using only an electronic sphygmomanometer may cause data deviations. Occupational stress surveys using the OSI-R scale are not consistent with other studies and may result in survey bias. In this study, only two sites of GR gene associated with hypertension were detected, respectively GR BCL1 and GR G678S. However, only GR BCL1 and the incidence of hypertension were statistically significant. In future, the sample size should be increased to enhance further research and analysis.

## Conclusion

In this study, 1280 desert petroleum workers were randomly selected by cluster sampling. Once exclusion criteria were applied, 1080 individuals were admitted into the cohort study. These workers were regularly followed for 2 years, using stringent measurement methods to determine the blood pressure and occupational stress of desert petroleum workers. After the follow-up, the hypertensive patients were screened out to analyze the effects of GR gene polymorphism and occupational stress on hypertension. The study found that the risk factors for hypertension were smoking, drinking, occupational stress,GG genotype and CG + GG genotype at GR BCL1 gene. Based on the above results, desert petroleum workers should pay attention to their own health, stop smoking and drinking, develop good habits, and control the occurrence of hypertension from the source. At the same time, the employer should improve the working environment, establish a reasonable system, give workers welfare treatment and humanistic care, relieve mental pressure in time, and create a harmonious and safe working environment for desert petroleum workers, improve their professional quality of life.

## Abbreviations

GCs: Glucocorticoids
GR: Glucocorticoid Receptor
HPA: Hypothalamic Pituitary Adrenal
ORQ: Occupational Roles Questionnaire
OSI-R: Occupational Stress Inventory (revised)
PRQ: Personal Resources Questionnaire
PSQ: Personal Strain Questionnaire
